# A CRISPR-Based Mutagenesis Strategy for Examining CLAG3 Helix 44 Contribution to Malaria Parasite Nutrient Uptake Channels

**DOI:** 10.3390/genes17040462

**Published:** 2026-04-15

**Authors:** Zabdi Gonzalez-Chavez, Mansoor A. Siddiqui, Sundar Ganesan, Sanjay A. Desai

**Affiliations:** 1 Laboratory of Malaria and Vector Research, National Institute of Allergy and Infectious Diseases, National Institutes of Health, Rockville, MD 20852, USA; 2 Biological Imaging Section, Research Technologies Branch, National Institute of Allergy and Infectious Diseases, National Institutes of Health, Bethesda, MD 20892, USA

**Keywords:** malaria, ion channels, nutrient uptake, saturation mutagenesis, CRISPR transfection, PSAC, ion channel selectivity

## Abstract

**Background:** Malaria parasites import essential nutrients from plasma into their host erythrocytes through the plasmodial surface anion channel (PSAC), a conserved ion and nutrient channel on the infected cell surface. A parasite-encoded ternary complex consisting of CLAG3, RhopH2, and RhopH3 determines PSAC activity, but the precise contributions of each member to formation of the nutrient uptake pore remains uncertain. **Methods:** Here, we devised a two-step CRIPSR transfection strategy to examine an amphipathic CLAG3 helix, termed α-helix 44 (α-H44), as a candidate pore-lining domain. **Results:** A CLAG3 truncation protein without α-H44 phenocopies a CLAG3 knockout line, suggesting a critical role of α-H44 in formation of the nutrient channel; CLAG3 restoration using a recodonized α-H44 restores PSAC activity fully. A saturation mutagenesis library that splits the helix into four sequential segments was devised and implemented. Two engineered mutants exhibit distinct PSAC phenotypes; their cultures failed to expand in a modified medium that approximates in vivo nutrient availability. **Conclusions:** These studies support a α-H44 role in channel permeation and block by a strain-specific inhibitor. Our strategy will enable saturation mutagenesis to determine how PSAC achieves its unique ion and nutrient selectivity and should help guide drug discovery against this antimalarial target.

## 1. Introduction

Malaria, a globally important cause of disease and mortality, is caused by parasites from the genus *Plasmodium*. These single-celled eukaryotes invade and replicate in vertebrate erythrocytes to evade host immune responses and access hemoglobin as an amino acid source. *Plasmodium falciparum*, the most virulent human pathogen, remodels its host cell through exported effector proteins that enable infected cell cytoadherence, immune evasion, virulence, and nutrient uptake [1,2,3,4,5]. While other *Plasmodium* spp. also carry out these functions via exported proteins, each species uses its own collection of surface-exposed effector proteins.

Nutrient uptake is an important exception as it is mediated by the conserved plasmodial surface anion channel (PSAC); this channel has been linked to three interacting proteins, CLAG3, RhopH2, and RhopH3, which are also conserved in all examined *Plasmodium* species [6,7,8,9]. Although molecular studies have provided much information about these proteins [5], the precise contributions of each protein to formation of the channel pore remain unclear. Unlike most channel proteins, each subunit has only one predicted transmembrane domain. Remarkably, these proteins are trafficked as a soluble ternary complex through multiple parasite developmental stages until shortly before its insertion into the host membrane [10], where the member proteins remain associated to form the functional nutrient uptake channel [11,12]. Protease susceptibility studies suggest that only CLAG3 is surface-exposed with a variable extracellular loop upstream of an amphipathic α-helix [8]. This α-helix 44 (α-H44, based on α-helix numbering from the N-terminus of CLAG3) has been proposed as a pore-lining helix based on segregation of hydrophobic and hydrophilic residues to opposite helix faces and on changes in PSAC selectivity in leupeptin-resistant mutant generated by in vitro selection [13].

Here, we devised and implemented a two-step CRIPSR transfection strategy to examine α-H44’s contribution to the nutrient channel pore through mutagenesis. An α-H44 truncation mutant and distinct mutants generated by our strategy provide independent evidence for a critical role of this helix in formation of the nutrient pore. The studies are consistent with a model where α-H44 lines the pore and contributes to recognition and permeation of essential nutrients that fuel parasite growth and replication.

## 2. Materials and Methods

### 2.1. In Vitro P. falciparum Culture

Wildtype Dd2 and KC5 *P. falciparum* clones and the transfected KC5 derivatives were cultured using O^+^ human erythrocytes from commercial sources (University of Virginia Blood Bank, Charlottesville, VA, USA). Cultures were maintained at 5% hematocrit, 37 °C in RPMI 1640 supplemented with 28.6 mM NaHCO_3_, 25 mM HEPES, 50 µg/mL hypoxanthine, 10 mg/L gentamicin, and 0.5% lipid-rich BSA (Albumax II, Thermo Fisher Scientific, Waltham, MA, USA or AlbumiNZ, MP Biomedicals, Irvine, CA, USA) under 90% N_2_, 5% CO_2_, and 5% O_2_. Transfection clones were maintained without drugs for selection markers after limiting dilution cloning.

### 2.2. DNA Transfection to Produce CLAG3 Truncation and α-H44 Mutants

The *H44trunc* clone was produced by CRISPR transfection using the *pUF1-Cas9* and *pL7-h44ko-bsd* plasmids with drug selection. The *pL7-h44ko-bsd* plasmid was prepared by subcloning of a synthetic 1259 bp fragment consisting of a 320 bp 5′ homology arm from the KC5 *clag3h* sequence, an in-frame recodonized sequence from Dd2 *clag3.1* encoding the hypervariable region and distal sequences up to α-H44, an optimal synthetic protospacer and PAM domain (5′-GCTAAGATACAACAACCCAG-cgg-3′; stop codon in red, PAM in lower case), recodonized downstream sequence, and a 288 bp 3′ homology arm from KC5 that ends with the native gene stop codon. Subcloning of this fragment and of a sgRNA targeting the KC5 *clag3h* gene (5′-CTATTAAAACTATGAACTGA-3′) were performed by In-Fusion cloning (Takara Bio, San Jose, CA, USA). DNA transfection was performed as described previously [5] using *pUF1-Cas9* and *pL7-h44ko-bsd* with selection using 1.5 µM DSM1 and 2.5 µg/mL blasticidin S until parasite outgrowth. Limiting dilution cloning yielded the *H44trunc* line.

*H44trunc* was then transfected with *pUF1-Cas9* and *pL7-NNK_library-hdhfr* to carry out the second transfection, which replaces the synthetic protospacer and PAM domain with either a recodonized sequence for the native α-H44 or a *NNK* library mutant. The *pL7-NNK_library-hdhfr* plasmids were prepared from *pL7-h44ko-bsd* prior to In-Fusion cloning of that plasmid’s sgRNA by replacing the *bsd* selection marker with the *hdhfr* selection marker [5]. The recodonized or mutant α-H44 domains were obtained as gBlocks (Integrated DNA Technologies, Coralville, IA, USA), with 6 consecutive codons using degenerate *NNK* bases for each of 4 α-H44 segments. These gBlocks and the sgRNA (5′-GCTAAGATACAACAACCCAG-3′) targeting the protospacer introduced in the first transfection were subcloned by In-Fusion cloning. After transfection as above, cultures were selected with 1.5 µM DSM1 and 2 nM WR99210. All experiments shown were performed with sequence-verified clones obtained by limiting dilution and the c-SNARF method [14].

### 2.3. Sorbitol Transport Assays

PSAC-mediated sorbitol uptake was tracked kinetically by following osmotic lysis of infected cells by recording 700 nm light transmittance through cell suspensions [15]. Indicated parasite clones were harvested from synchronous trophozoite-stage cultures, enriched by the Percoll–sorbitol method, washed and resuspended in 140 mM NaCl, 20 mM HEPES, and 0.1 mg/mL bovine serum albumin (BSA), pH 7.4 with NaOH. Sorbitol uptake was initiated by addition of 20 volumes of lysis solution (280 mM sorbitol, 20 mM HEPES, 0.1 mg/mL BSA, pH 7.4) to yield a 0.1% hematocrit cell suspension. Osmotic lysis at 37 °C was then tracked by measuring 700 nm light transmittance (DU640 or DU800 spectrophotometer with Peltier temperature control, Beckman Coulter, Brea, CA, USA). Where indicated, ISPA-28 was added from a DMSO stock solution to the lysis solution. Lysis halftimes were determined by interpolation using locally developed scripts; inhibitor dose–response data were fitted to the sum of two Lagmuir isotherms as:

*P* = *a*/(1 + *x*/*b*)) + (1 − *a*)/(1 + *x*/*c*)) (1)

where *P* represents the normalized sorbitol permeability with ISPA-28 at *x* concentration and *a*, *b*, and *c* are fitted constants.

### 2.4. Immunoblots

Trophozoite-stage cultures were Percoll–sorbitol enriched, washed, and resuspended at 5% hct in 150 mM NaCl, 20 mM Na_2_HPO_4_, 0.6 mM CaCl_2_, 1 mM MgCl_2_, pH 7.4. Pronase E (Sigma Aldrich, St. Louis, MO, USA), where used, was freshly added to intact cells at 1 mg/mL for a 1 h incubation at 37 °C prior to inactivation and removal with washes in buffer with 2 mM PMSF. Hypotonic lysis of cells used 7.5 mM Na_2_HPO_4_, 1 mM EGTA, pH 7.2, and incubation at room temperature for 30 min followed by ultracentrifugation (100,000× *g*, 1 h, 4 °C). The pellet was subjected to alkaline extraction after resuspension in 100 mM Na_2_CO_3_, pH 11 for 30 min before ultracentrifugation (100,000× *g*, 1 h, 4 °C) to harvest an integral membrane protein fraction. All samples were subjected to SDS-PAGE after solubilization in a modified Laemmli sample buffer containing 6% SDS (4 to 15% Mini-Protean TGX gel, Bio-Rad, Hercules, CA, USA). Separated proteins were then transferred to nitrocellulose prior to blocking with 3% skim milk in TBS-T (20 mM Tris, 150 mM NaCl, 0.1% Tween20 pH 7.5) for 1 h at room temperature, then incubated overnight at 4 °C with primary antibody (CLAG3 antibody, prepared locally in mice [7]; 1:3000 dilution), washed three times before the secondary antibody was applied (HRP-conjugated anti-mouse IgG, 1:3000, Sigma Aldrich) for 3 h at 4 °C. Clarity Western ECL Substrate (Bio-Rad) and the Amersham Imager 680 was used to obtain images. Exposure times varies from 1–30 s. Sample loading was assessed by ImageJ version 1.54 (https://imagej.net/ij/, accessed on 5 March 2026) quantification of hemoglobin band intensity after Ponceau S staining.

### 2.5. Indirect Immunofluorescence Assays (IFA)

Protein localization was performed using indirect immunofluorescence confocal microscopy with thin smears prepared from parasite cultures resuspended at 40–50% hematocrit. The smears were briefly air dried and then fixed in 90:10 acetone:methanol at −30 °C. Fixed smears were then blocked with 3% BSA in PBS overnight, washed, and incubated overnight with primary antibodies in 3% BSA (mouse anti-CLAG3 [7], 1:200 dilution; rabbit anti-RhopH3 [8], 1:350 dilution). After washing in 3× PBS, secondary antibodies were applied (Alexa Fluor 594 anti-mouse and Alexa Fluor 488 anti-rabbit, at 1:1000 dilution each; Thermo Fisher, Waltham, MA, USA). Slides were mounted with coverslips using ProLong Gold Antifade mountant with DAPI (4′,6-diamidino-2-phenylindole). Confocal images were acquired on a DMI8-SP8 WLL-FALCON confocal microscope (Leica Microsystems, Buffalo Grove, IL, USA) with fixed acquisition settings at 1024 pixel format using 63× NA 1.4, oil immersion objective. Images were processed using Imaris 10.2.0 image analysis software (Oxford Instruments, Abington, UK). Images were deconvoluted using Huygens Professional 25.10.1 software (SVI Images, Hilversum, The Netherlands) using standard and conservative iteration methods. Colocalization was assessed using the Pearson’s correlation coefficient [16].

### 2.6. Parasite Expansion in PGIM

Parasite growth in PGIM was measured using a SYBR Green I-based assay with normalization to growth in matched cultures using standard RPMI 1640-based media. Synchronous ring-stage cultures were seeded at 2.5% hematocrit and 0.15% parasitemia in 96-well microplates in 200 µL of standard RPMI 1640 medium, PGIM, or PGIM supplemented with 20 µM chloroquine. Media was replaced after 72 h cultivation. After 5 days, 100 µL medium was removed and replaced with 20 mM Tris pH 7.5, 10 mM EDTA, 1.6% Triton, 0.016% saponin, and 4× SYBR Green I nucleic acid stain (Thermo Fisher). After a 20 min incubation, fluorescence was measured to estimate parasite DNA production (excitation, 485 nm; emission 528 nm; Synergy Neo 2 plate reader, Biotek, Winooski, VT, USA).

### 2.7. Statistics

All numerical data were tabulated and plotted as mean ± S.E.M. from three or more trials. Statistical significance was calculated in SigmaPlot 16.0 (Grafiti LLC, Palo Alto, CA, USA) using unpaired Student’s *t*-test or one-way ANOVA tests as appropriate. Post hoc pairwise comparisons were made as described.

## 3. Results

### 3.1. A Two-Step Transfection Strategy to Facilitate Site-Directed Mutagenesis and Evaluation of α-Helix 44′s Role

**Figure 1:**
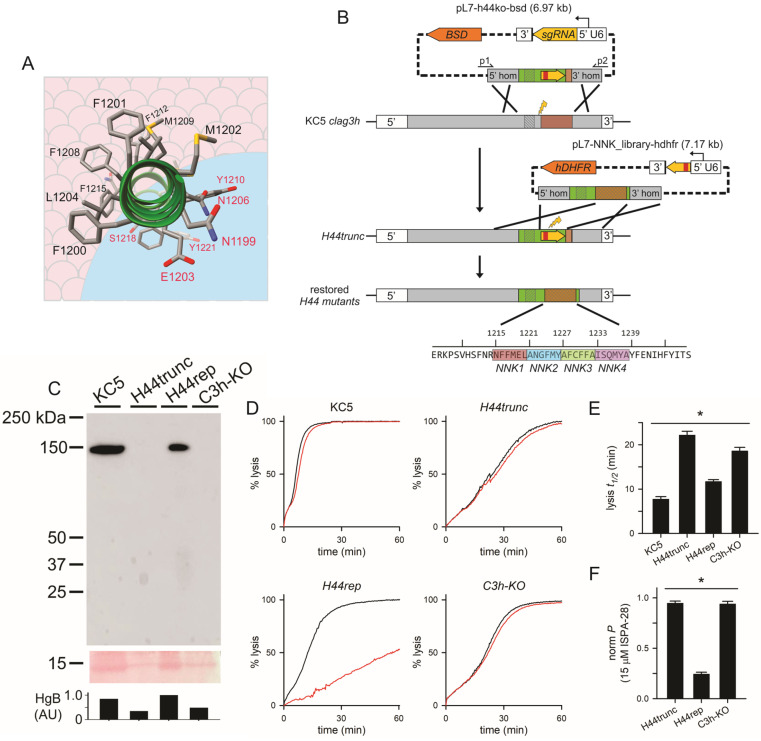
A robust strategy for mutagenesis of the CLAG3 α-H44. (**A**) Schematic showing α-H44 as a pore-lining helix of CLAG3. A helical wheel projection of α-H44 is shown on a channel pore to illustrate the segregation of hydrophobic and polar/charged residues to lipidic and aqueous faces (tan and blue color backgrounds, respectively). The α-H44 is shown enface from its N-terminus as it enters the membrane from the extracellular space. Note that charged and polar residues face the pore while hydrophobic residues (esp. phenylalanine side chains) face the lipid bilayer. (**B**) Ribbon schematic showing the two-step CRISPR transfection strategy for α-H44 mutagenesis. *H44trunc* is generated by the first transfection to introduce a recodonized upstream CLAG3 including the HVR of Dd2 CLAG3.1 (green) and a high-scoring protospacer with a stop codon (yellow arrow with red vertical hash). The second transfection targets this protospacer to restore a full-length CLAG3 with desired mutations over α-H44. The wild-type α-H44 sequence, restored in the *H44rep* clone, is shown at the bottom with the four *NNK* segments highlighted. (**C**) Immunoblot of indicated lines, showing that *H44trunc* and *C3h-KO* are not recognized by an α-CLAG3 antibody directed to the protein’s C-terminus [7] and that *H44rep* carries a restored full-length protein comparable to the wild-type parent. Bottom gel, Ponceau S staining of the blot. Bar graph, ImageJ quantification of primary hemoglobin band (HgB, ~15 kDa); sample loading varied up to 3-fold. (**D**) Sorbitol-induced osmotic lysis kinetics for indicated lines without and with 15 µM ISPA-28 (black and red traces, respectively). Osmotic lysis is slower in *H44trunc* and *C3h-KO* due to CLAG3 truncation or knockout; ISPA-28 block is present only in *H44rep* as it carries a full-length CLAG3 with the Dd2 CLAG3.1 HVR sequence. (**E**) Mean ± S.E.M. osmotic lysis half-times (*t_1/2_*) interpolated as the time to 50% lysis in kinetic experiments such as shown in panel (**D**). Sorbitol permeability is inversely proportional to the lysis *t_1/2_*. (**F**) Mean ± S.E.M. sorbitol permeability in the presence of 15 µM ISPA-28, normalized to matched control lysis kinetics without ISPA-28. *, *p* < 0.001, one-way ANOVA tests.

The CLAG3 α-helix 44 (α-H44), as identified in cryo-EM structure of the pre-membrane embedded RhopH complex [10], is the only high-scoring predicted transmembrane domain in CLAG3. This helix is amphipathic, with hydrophobic and hydrophilic residues on opposite faces. The cryo-EM structure revealed a preponderance of phenylalanine side chains on the hydrophobic face (Figure 1A, [10]), enabling stable interaction with the lipid bilayer. Moreover, a leupeptin-resistant PSAC mutant, which carries a single mutation localizing to α-H44, has altered selectivity for many solutes [13]. These observations suggest that α-H44 is a pore-lining helix (Figure 1A).

To experimentally evaluate α-H44 role in PSAC activity, we devised a two-step transfection strategy that increases CRISPR-mediated homology repair and maximizes production of site-directed α-H44 mutants (Figure 1B). For these transfections, we selected the KC5 parasite wild-type clone because it has a single *clag3* gene, termed *clag3h* [17], in contrast to the two copies in most other lines; KC5 has also been successfully used for CLAG3 modifications previously [5,18].

In the first step of our strategy, the *clag3h* last exon is targeted for replacement using CRISPR/Cas9 and an sgRNA targeting a conserved *clag3* sequence with a cut site 4555 bp from the gene start ATG. The homology arms were selected to cover α-H44 and an upstream variant region of *clag3* known as the hypervariable region (HVR). One variant, carried by the Dd2 clone’s *clag3.1* gene, encodes an HVR that confers potent PSAC block by ISPA-28, a small molecule inhibitor from high-throughput screens [7]. In our strategy, successful homology-directed repair replaces the KC5 HVR with that of Dd2 *clag3.1*, yielding ISPA-28 sensitivity and enabling selection for PSAC mutants, as reported previously [5,15]. This transfection step also replaces the KC5 α-H44 with an unnatural protospacer and protospacer adjacent motif (PAM) that can be targeted in the second transfection (Figure 1B, transfection with *pL7-h44ko-bsd*). We designed this unnatural protospacer using SpCas9 protospacer position preferences to produce near-maximal on-target efficiency cleavage and editing to introduce modifications in the second transfection [19]. Our design includes a stop codon in this protospacer to yield a truncated CLAG3 protein after the first transfection (*H44trunc*, Figure 1B); we envisioned that restoration of the full-length protein in the second transfection would permit selection of gain-of-function α-H44 mutants. The second CRISPR transfection targets the unnatural protospacer and replaces it with either a recodonized sequence encoding the original α-H44 (*H44rep*) or a randomized mutant sequence to evaluate effects on PSAC-mediated transport (Figure 1B, transfection with *pL7-NNK_library-hdhfr*). Because mutagenesis over all 24 residues in the α-H44 motif involves an excessively large collection of possible mutants (20^24^ permutations), we divided α-H44 into 4 segments with 6 codons each and used a degenerate *NNK* sequence for each codon; the use of the K degenerate base (G or T) in the 3rd “wobble” position minimizes introduction of stop codons [20]. This approach also has the advantage that it permits identification of specific segments of α-H44 with greater controlling effects on solute permeation or selectivity. We termed the libraries of mutants for each α-H44 segment *NNK1* to *NNK4*, which encode groups of 6 residues starting from the extracellular end of α-H44 to the intracellular face of the putative pore, respectively (restored H44 mutants, Figure 1B).

DNA transfection and limiting dilution successfully yielded the desired *H44trunc* clone, which was then subjected to the second CRISPR editing to produce the *H44rep* clone. As predicted, the truncated CLAG3 protein in *H44trunc* is not recognized by an anti-CLAG3 antibody that targets the protein’s C-terminus but the restored full-length protein of *H44rep* is recognized as a single band comparable to that of the KC5 parent (Figure 1C). A recently generated CLAG3 knockout line, *C3h-KO*, was used as a knockout control for comparison and is also not recognized by this antibody (Figure 1C, [5]).

### 3.2. α-H44 Is Required for CLAG3-Associated Solute Transport

We next examined PSAC activity in these transfectant lines and used a quantitative transmittance assay to track uptake of sorbitol, a sugar alcohol that is impermeant to uninfected erythrocytes but has a high uptake via PSAC after infection [15]. As observed with other wild-type lines, KC5 exhibited rapid sorbitol uptake and osmotic lysis with a halftime, *t_1/2_* of 7.8 ± 0.5 min (Figure 1D,E). *H44trunc* exhibited a slower uptake comparable to *C3h-KO*, indicating that loss of α-H44 and distal CLAG3 segments compromises channel function equivalent to complete CLAG3 loss. Interestingly, these lines still undergo sorbitol uptake and osmotic lysis, indicating that CLAG3 knockout does not fully abolish PSAC activity. Current models suggest that CLAG paralogs encoded by *clag* genes on other chromosomes in *P. falciparum* serve overlapping roles and can also form channels on the host membrane [5]; this model is supported by a dramatically increased copy number of *clag2*, a paralog on chromosome 2, when C3h-KO is subjected to nutrient stress [21]. Transfection of *H44trunc* to restore a full-length CLAG3 in the *H44rep* line restored sorbitol uptake to levels approaching that of the wild-type parent (Figure 1D,E). The modestly lower sorbitol uptake in *H44rep*, apparent as a somewhat greater *t_1/2_* (Figure 1E) may result from production of a CLAG3 chimera that combines the upstream KC5 with distal Dd2 sequences or from epigenetic changes in *clag3* expression as a byproduct of DNA transfection, as observed previously [22]. Thus, the C-terminal CLAG3 region, including α-H44, is essential to this protein’s contribution to the channel pore.

We examined inhibition of sorbitol uptake by ISPA-28 to further validate the role of the Dd2 *clag3.1* HVR sequence in mediating block by this strain-specific PSAC inhibitor. As observed previously, KC5 channels are not inhibited by this agent (Figure 1D, red trace). *H44rep* exhibited strong inhibition, as predicted for a full-length CLAG3 protein carrying the HVR sequence from Dd2 *clag3.1* (Figure 1D,F). The truncated or absent CLAG3 proteins in *H44trunc* and *C3h-KO* yielded channels that are not detectably inhibited (Figure 1D,F). These findings support a model where CLAG3 defines the channel pore and the HVR region contributes to the ISPA-28 binding site on the channel.

### 3.3. Transfection Yields a Diverse Collection of α-H44 Mutants

To explore saturation mutagenesis on α-H44, we cloned the homology repair cassette carrying pools of degenerate mutants *NNK1* through *NNK4* as described above into the *pL7-NNK_library-hdfr* plasmid. These separate mutant pools were then used in CRISPR transfections into the *H44trunc* line with the aim of generating many clones with distinct mutations over each of the four α-H44 segments. Our initial trials yielded a disappointingly small number of distinct mutants upon sequencing individual clones, obtained by limiting dilution shortly after outgrowth of transfectant parasites. Although one explanation is that many mutations in this critical helix may be lethal to parasite survival, we note that this hypothesis predicts marked enrichment for truncation mutants that carry “TAG” stop codons that remain possible with *NNK* libraries. Such truncation mutants would produce a truncated CLAG3 protein nearly identical to that of *H44trunc* and are expected to expand normally. As enrichment for these truncation mutants was not observed, the small number of recovered mutants presumably results from the low transfection efficiency in *P. falciparum* combined with so-called “founder effects” during parasite outgrowth [23]. With improved transfection technologies and optimization of transfection protocols in microplate formats [24,25], it should be possible to recover and characterize many α-H44 variants using this approach.

**Figure 2:**
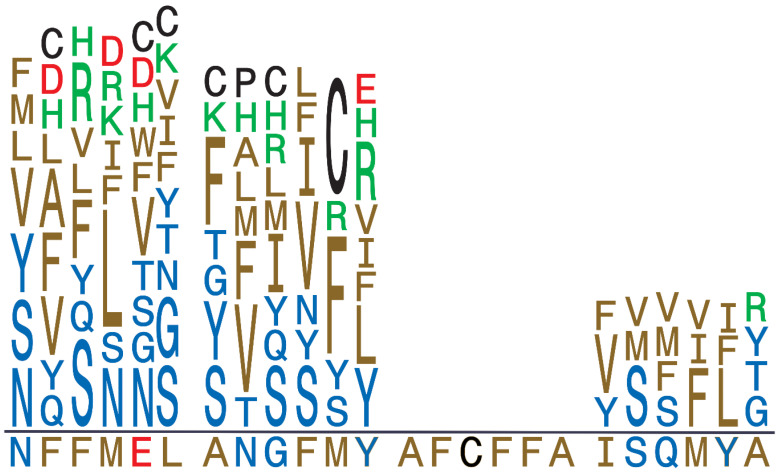
Sequence logo showing position-specific substitutions in integrants obtained over the 4 *NNK* segments (12, 11, and 4 clones each for *NNK1*, *NNK2*, and *NNK4*). Residue color codes: hydrophobic, gold; polar, blue, negatively charged, red; positively charged, green; special residues, black. Residue heights indicate the number of integrants carrying a specific replacement. The wild-type sequence is shown below the horizontal line.

To obtain proof-of-principle results, we next performed transfections using single *NNK* variants by recovering plasmid from individual *E. coli* library colonies. We selected the *NNK1*, *NNK2*, and *NNK4* mutant libraries and used DNA sequencing to exclude bacterial colonies carrying stop codons and performed transfections. We successfully obtained 27 distinct integrants covering these three α-H44 segments. Figure 2 shows the residues incorporated at each α-H44 position with residues color-coded according to amino acid property and letter height indicating the number of integrants with that specific residue substitution. Most α-H44 positions were successfully replaced with hydrophobic, polar, and both positively and negatively charged residues, indicating that this approach is well-suited for examining effects of amino acid properties at each position along the putative pore-lining helix.

### 3.4. Two NNK Mutants with Distinct Phenotypes

**Figure 3:**
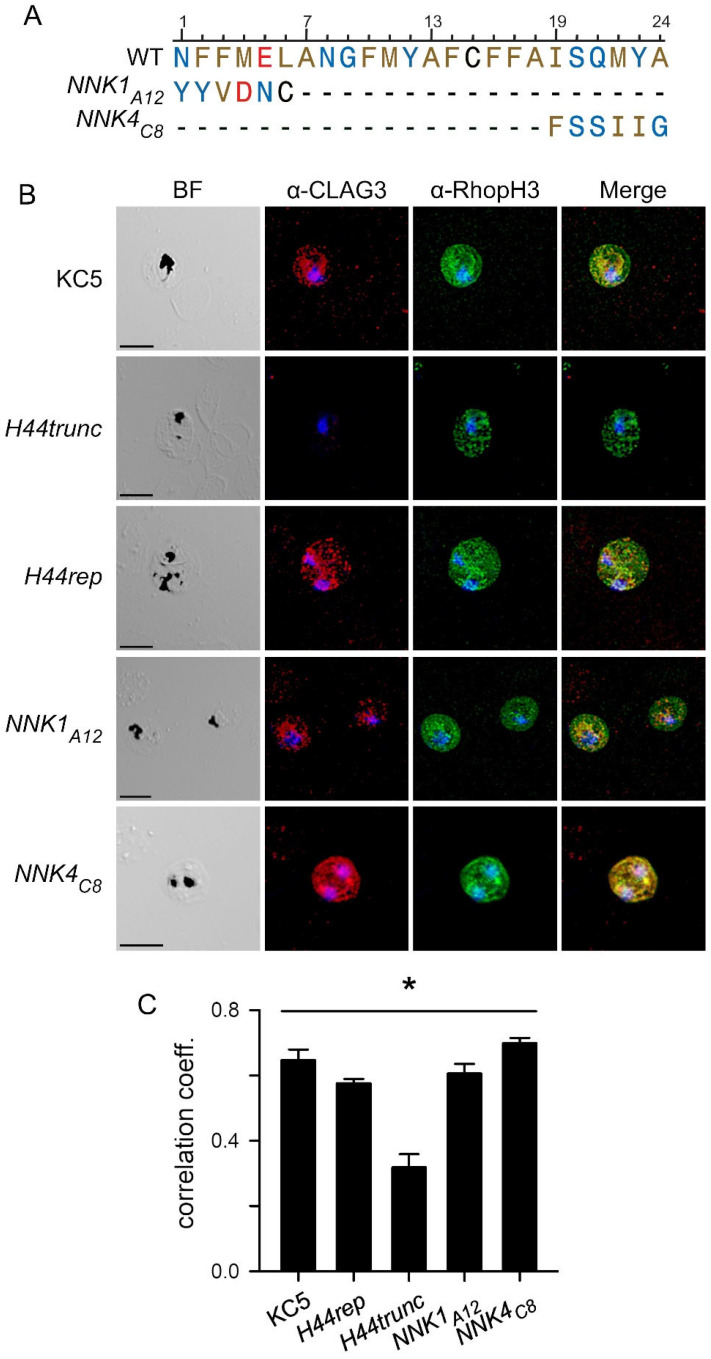
Two *NNK* mutants with distinct phenotypes. (**A**) α-H44 sequence alignment for wild-type lines (WT) and indicated mutant clones. Residues are color-coded according to amino acid property as in Figure 2. Dash indicates position identity with the WT sequence. Note that both mutant clones have mutations at most positions but that the net physicochemical properties of residues over their *NNK* segment are similar to those of the wildtype. (**B**) Confocal microscopic images from indirect immunofluorescence assays (IFA) of trophozoite-stage infected cells from indicated parasite clones with α-CLAG3 and α-RhopH3 antibodies. Note that *H44trunc* lacks CLAG3 and that each other clone has CLAG3 and RhopH3 signals in the host compartment of infected cells; signal is evident at the host cell surface. Scale bars, 5 µm. Images shown are representative of >50 cells for each clone. (**C**) Pearson’s correlation coefficients for colocalization of CLAG3 and RhopH3 from IFA images. * *p* < 0.001, one way ANOVA, *n* = 10–11 cells each.

We next examined the precise contributions of α-H44 in trafficking, membrane insertion at the host cell surface, and formation of the nutrient channel pore with two separate *NNK* mutants. We selected one clone each incorporating mutations in *NNK1* and *NNK4* to evaluate phenotypes resulting from mutations at opposite ends of the α helix and engineered parasite transfectants *NNK1_A12_* and *NNK4_C8_* (Figure 3A). These clones carry substitutions of wild-type residues at 5 or 6 of the 6 residues covered by individual *NNK* segment libraries, but both also represent relatively conservative changes as the net charge and overall hydrophobicity of the corresponding segment is preserved; both lack stop codons and expected to produce full-length CLAG3 protein. We confirmed production of full-length protein in both transfectant clones with indirect immunofluorescence assays. These imaging studies established that full-length CLAG3 is detected in erythrocytes infected with KC5 and *H44rep* but is undetectable in *H44trunc* (Figure 3B), paralleling the immunoblotting results (Figure 1C). Consistent with direct contribution to PSAC formation at the host membrane, CLAG3 and RhopH3 were exported into host cytosol and colocalized at the erythrocyte surface in KC5 and *H44rep*. A clear CLAG3 signal was also present in *NNK1_A12_* and *NNK4_C8_*, with export into the host cell and RhopH3 colocalization at the erythrocyte surface (Figure 3B). These results indicate that the introduced α-H44 mutations do not compromise CLAG3 transfer from the prior host cell or export and trafficking to the host cell.

**Figure 4:**
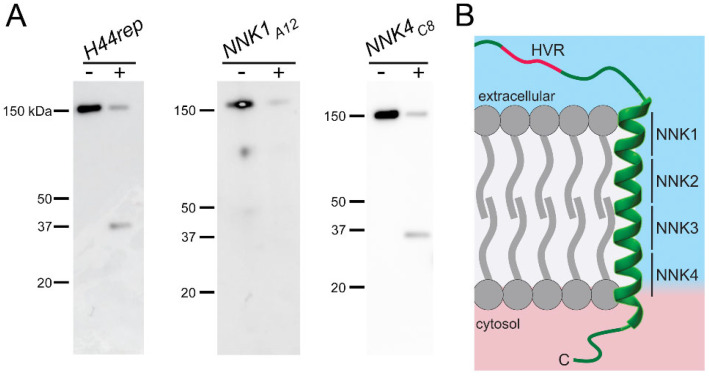
Immunoblotting establishes full-length CLAG3 and evaluates susceptibility to extracellular protease. (**A**) α-CLAG3 immunoblots without and with pronase E treatment as indicated. The band at ~160 kDa reflects full-length CLAG3; a ~35 kDa cleavage product in *H44rep* and *NNK_C8_* represents a C-terminal fragment released by proteolysis in the extracellular HVR. (**B**) Schematic showing the proposed model where α-H44 lines a water-filled pore and the HVR is in a protease-susceptible extracellular loop. The *NNK* segments used to produce mutant parasite clones are marked to highlight that *NNK1* is at the extracellular end of the helix.

Immunoblots then showed both mutant clones carry the ~160 kDa full-length protein (Figure 4A). Prior studies have used treatment of intact infected erythrocytes with extracellular protease to define a CLAG3 extracellular loop that can be cleaved by various proteases [26]. Such studies have established that CLAG3 is integral to the host membrane, determined that the protease-susceptible extracellular loop corresponds to the hypervariable region (HVR, Figure 4B), and provided independent evidence for a direct contribution of CLAG3 to the functional nutrient channel through linkage analysis [26]. We therefore used protease treatment of cells infected with our transfectants to examine membrane insertion and mutant CLAG3 surface presentation (Figure 4A). Protease treatment of *H44rep*, which expresses a full-length chimeric protein with an unmodified α-H44, yielded a ~37 kDa cleavage product that corresponds to the size of a C-terminal fragment released upon cleavage in the HVR; the ~120 kDa fragment upstream of the HVR is not recognized by this antibody raised against the CLAG3 C-terminus [7]. This transfection control, therefore, matches results with wild-type parasite lines [26]. *NNK4_C8_* also yielded a comparable C-terminal cleavage product with a modestly faster migration of ~35 kDa, suggesting that mutations in α-H44 may alter the specific residues within the HVR that are most protease susceptible. Several attempts with the *NNK1_A12_* clone failed to uncover a clear cleavage product (Figure 4A). This could reflect compromised insertion of the mutant α-H44 in the host membrane or an altered presentation of the extracellular HVR loop that renders it less susceptible to external proteases. As the *NNK1* segment would be the first to insert into the host membrane in most models describing two-state protein insertion of soluble proteins into lipid bilayers [27,28], mutations in *NNK1* may have the greatest effect on faithful insertion and presentation of epitopes at the extracellular channel face.

**Figure 5:**
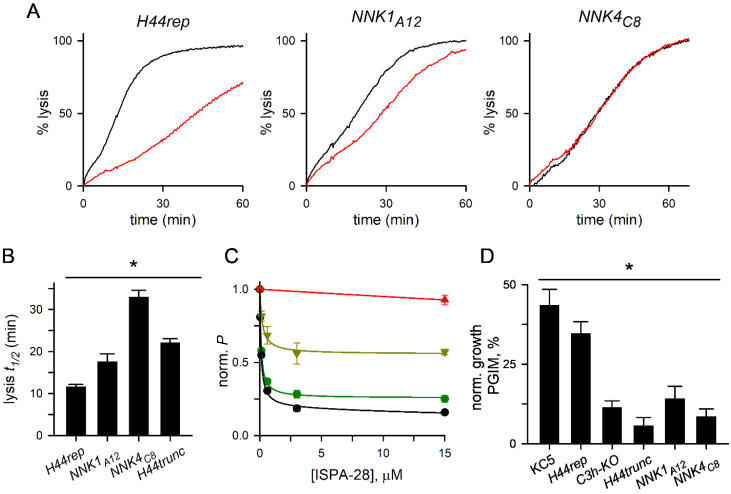
PSAC phenotypes and in vitro expansion in nutrient-limiting media. (**A**) Osmotic lysis kinetics for indicated lines in sorbitol without and with 15 µM ISPA-28 (black and red traces, respectively). (**B**) Mean ± S.E.M. lysis half-times (*t_1/2_*) of indicated lines. * *p* < 0.001, Kruskal–Wallis one-way ANOVA. (**C**) ISPA-28 dose–response for PSAC block, determined from lysis kinetics, for wild-type Dd2 (black circles), H44rep (green circles), NNK_A12_ (gold triangles), and NNK_C8_ (red triangles). H44rep reproduces the ISPA-28 block of Dd2 channels while NNK_A12_ exhibits partial block at high [ISPA-28] and NNK_C8_ channels are not blocked. *p* < 0.001, one way ANOVA. (**D**) Mean ± S.E.M. expansion of indicated lines in PGIM, normalized to matched cultures in standard RPMI 1640-based medium. * *p* < 0.001, one way ANOVA; H44rep growth is indistinguishable from that of KC5 while the NNK mutants match growth of the C3h-KO and H44trunc knockout lines in pairwise comparisons, Holm–Sidak method.

We next tracked sorbitol uptake in these mutants to assess the role of α-H44 in permeation through PSAC. Although the full-length CLAG3 proteins in *H44rep*, *NNK1_A12_*, and *NNK4_C8_* are identical except for the conservative mutations introduced in α-H44, the resulting channel activities differed markedly. Sorbitol uptake differed between these clones significantly, as quantified using lysis halftimes (Figure 5A,B). 

Although the mean lysis halftime for *NNK4_C8_* was nearly twice that of *NNK1_A12_* (Figure 5B)*,* post hoc comparison of these two α-H44 mutants by the Dunn’s method did not reach statistical significance. Uptake in *NNK1_A12_* was intermediate between *H44rep* and *H44trunc*, suggesting formation of a functional pore with reduced sorbitol permeability. ISPA-28 block also differed between the two α-H44 mutants even though both carry the responsible HVR from the Dd2 CLAG3.1 protein. While ISPA-28 dose–response experiments confirmed that block of *H44rep* channels is comparable to that of the wild-type Dd2 (Figure 5C), *NNK1_A12_* channels were only partially blocked by a high 15 µM ISPA-28 and *NNK4_C8_* were not measurably blocked (Figure 5A,C). Thus, although the Dd2 CLAG3.1 HVR sequence is required for ISPA-28 block of channels, changes in the α-H44 sequence control ISPA-28 affinity and completeness of channel block.

We also examined growth rates of these mutants in PGIM, a modified medium with lower, more physiological levels of key nutrients acquired via PSAC [15]. Parasites with wild-type nutrient uptake expand more slowly in PGIM, at growth rates that are 25–40% of those in nutrient-rich RPMI 1640 medium typically used for *P. falciparum* cultivation; this slower rate matches expansion rates in pooled human serum, suggesting that PGIM more faithfully represents in vivo parasite growth [15]. While expansion of *C3h-KO* is indistinguishable from that of its wild-type KC5 parent in standard RPMI 1640-based medium, it fails to propagate in PGIM because PSAC-mediated nutrient uptake is insufficient (Figure 5D, [5]); extended PGIM selection of *C3h-KO* led to a dramatic 18-fold increase in *clag2* copy number, suggesting the CLAG2 paralog expressed from the *P. falciparum* chromosome 2 compensates for CLAG3 knockout through upregulation [21].

We compared PGIM expansion of our transfectant lines and normalized growth to each line’s propagation in standard RPMI 1640-based medium. As seen previously, KC5 and the *H44rep* line, which exhibit wild-type nutrient transport, grew at 35–45% of their rates in RPMI 1640 (Figure 5D); *C3h-KO* and the *H44trunc* line, lines with identically slowed sorbitol permeabilities (Figure 1E), grew at much lower rates in PGIM. Although *NNK1_A12_* and *NNK4_C8_* have distinct channel properties, both were found to have similarly low expansion in PGIM (Figure 5D). These lines provide independent evidence for a direct link between PSAC-mediated uptake of nutrients and growth of the intracellular parasite.

## 4. Discussion

During their intracellular growth and replication within erythrocytes, malaria parasites use the conserved plasmodial surface anion channel (PSAC) to acquire essential nutrients from host plasma. Once assumed to reflect upregulated host ion channels [29,30,31,32], linkage analysis and DNA transfection studies have linked this channel to three conserved parasite proteins that are exported and assembled into a higher-order complex at the erythrocyte membrane [7,8,9]. Nevertheless, lack of homology to known ion channels and a paucity of predicted transmembrane domains in the three subunits have raised questions about how these proteins mediate formation of functional channels. An amphipathic α helix, α-H44, may define the channel pore based on segregation of hydrophilic residues to one face (Figure 1A) and a leupeptin-resistant mutant with marked changes in channel properties that result from a single mutation in this helix [13]. Here, we devised and implemented a two-step CRISPR transfection strategy to examine α-H44 role. Our studies support a critical role of this helix as CLAG3 truncation in α-H44 phenocopies CLAG3 knockout and two α-H44 mutants we generated exhibit distinct PSAC properties.

CLAG3 expressed by *NNK4_C8_* traffics normally to the host membrane and colocalizes well with RhopH3 (Figure 3). It remains susceptible to extracellular protease (Figure 4A). At the same time, *NNK4_C8_* has transport and growth properties most similar to *H44trunc*, a functional CLAG3 knockout (Figure 5). Thus, the α-H44 mutations in NNK4C8 yield a full-length CLAG3 protein that traffics normally but is entirely nonfunctional. In light of α-H44 amphipathicity with hydrophobic residues on one face and hydrophilic residues on the other helix face, the most conservative explanation for this finding is that α-H44 directly contributes to formation of the channel pore. The mutations in the *NNK4_C8_* α-H44 appear to collapse, block, or otherwise distort the pore to abolish transport through channels composed of one or more CLAG3 subunits. Conclusive evidence for this model may require an atomic resolution structure of the membrane-embedded ion channel.

We propose that α-H44 may define a water-filled pore through which ions and nutrients cross the host cell membrane. We also propose that this helix contributes to the channel’s selectivity filter. Selectivity filters in ion channels interact with dissolved solutes that enter the channel pore and dictate which ones are permitted to pass through the pore. Our model parallels those proposed for many cation channels, where pore-lining helices have been clearly defined through molecular and structural studies [33,34,35]; in these channels, specific residues have been convincingly implicated in selection of ions for permeation through the pore [36,37]. Interestingly, selectivity filters are less well-defined for most anion channels [38,39]; ClC chloride channels, for example, lack conventional selectivity filters as mutations in many regions of the protein alter selectivity and permeation of ions [40].

Although anions have the highest permeability of all solutes through PSAC, this channel’s unusual properties suggest key differences from the above anion channels. PSAC’s component subunits, CLAG3, RhopH2, and RhopH3, lack homology to known mammalian channels have only one predicted transmembrane domain each. More fundamentally, it has an unprecedented pattern of selectivity to solutes. Aside from high flux of monovalent anions, it is also permeant to bulky organic solutes and organic cations, with a remarkably high permeability to the guanidinium cation, C(NH_2_)_3_^+^ [41]. Despite this promiscuity, the small Na^+^ cation has a remarkably low flux through PSAC, with exclusion estimated at 10^6^-fold relative to Cl^−^; this level of exclusion for a single small, monovalent ion is unmatched amongst broad selectivity channels studied to date [42,43,44]. Another unusual feature is PSAC’s remarkably low single channel conductance of 20 pS in molar Cl^−^ solutions as broad selectivity channels permeant to bulky organic solutes typically have much higher throughput rates [45]. This low conductance may reflect a requirement for ion dehydration prior to flux through the channel, as implicated by inverse ion size-permeability relationship for both anions and cations [41]. The observed faster permeation of large ions than of small ions is surprising because we assume that small ions should more easily navigate a narrow pore. Energy barrier considerations reconcile this as this weakest theoretical binding site does not compensate for the greater Gibbs free energy lost in dehydrating small ions [46].

Our two-step transfection strategy should enable facile examination of PSAC’s unusual properties and help define the pore and selectivity filter(s) of this important parasite ion channel. There are several key advantages of our strategy. In the first transfection, we introduced large, recodonized homology arms on both sides of the α-H44 segment to facilitate efficient homology-directed repair after Cas9-mediated cleavage, increasing survival of successful transfectants [47]. The first transfection also introduces the upstream hypervariable region from Dd2 CLAG3.1 to confer ISPA-28 block, a feature that can be used for in vitro selection of integrants through osmotic lysis approaches that remove infected cells whose channels are not blocked [15]. It also replaces the α-H44 segment with a high-scoring synthetic protospacer that includes an in-frame stop codon, which we engineered using a study of *Sp*Cas9-mediated DNA cleavage efficiency of all four possible bases at each position in the protospacer sequence [19]; thus, this sequence approximates the best possible on-target efficiency score for any protospacer sequence. Importantly, it has negligible similarity to any 20-mer sequence in the *P. falciparum* genome, ensuring efficient cleavage and repair of the target site without unwanted off-target edits at other genomic sites [48]. Finally, the small insert size required in the second CRISPR transfection and comparable lengths of the α-H44 insertion and the protospacer deletion element have been empirically found to be optimal for homology-directed repair in the parasite genome [47].

We envision that our strategy will be combined with in vitro selection of transfection pools to enrich for the most interesting PSAC phenotypes in future studies. Gain-of-function mutants can be selected by use of low or physiological levels of key nutrients, as exemplified by the PGIM medium we used. For example, growth of mixed cultures in PGIM supplemented with an allele-specific inhibitor forced CLAG3 switching to isoforms that are inhibitor-resistant [15]; similar studies with mutant *NNK* transfection pools may uncover residues that define the inhibitor binding pocket. Interestingly, *P. falciparum* can be cultivated in sucrose-based media with low [Na^+^], suggesting that it is possible to select for PSAC mutants with high Na^+^ permeability [49]. Successful identification of such mutants could address how PSAC achieves its unprecedented Na^+^ exclusion and have fundamental implications for a broader understanding of ion channel permeation.

## Data Availability

The original contributions presented in this study are included in the article. Further inquiries can be directed to the corresponding author.

## Abbreviations

The following abbreviations are used in this manuscript:

α-H44	Alpha helix 44
CLAG2	Cytoadherence-linked antigen, expressed from chromosome 2
CLAG3	Cytoadherence-linked antigen, expressed from chromosome 3
DAPI	4′,6-diamidino-2-phenylindole
HVR	Hypervariable region
PAM	Protospacer adjacent motif
PSAC	Plasmodial surface anion channel
